# Dietary Intake of Plant-Based Meat Consumers—A Cross-Sectional Analysis Based on the National Diet and Nutrition Survey (Years 2014–2019) in the United Kingdom

**DOI:** 10.1016/j.cdnut.2026.109401

**Published:** 2026-06-17

**Authors:** Nicole Neufingerl, Laurine Ballintijn, Sander Biesbroek, Anne J Wanders

**Affiliations:** 1 Nutrition & Health Department, Unilever Foods Innovation Center, Wageningen, The Netherlands; 2 Division of Human Nutrition and Health, Wageningen University and Research, Wageningen, The Netherlands

**Keywords:** plant-based meat, meat alternatives, dietary intake, nutrient adequacy, United Kingdom, diet transition

## Abstract

**Background:**

Current knowledge on the quality of dietary intakes from plant-based meat (PBM)–containing diets is mainly based on theoretical modeling studies, whereas empirical evidence from actual consumption data is scarce.

**Objectives:**

This study aimed to investigate dietary intakes of PBM consumers compared with nonconsumers using real-life United Kingdom consumption data.

**Methods:**

Intakes of adults (18–65 y) from the National Diet and Nutrition Survey Rolling Program (2014–2019) in the United Kingdom were calculated based on a 4-d dietary diary. Nutrient intakes were expressed as percentages of nutrient-specific dietary reference values (DRVs), and Mann–Whitney *U* tests were performed to test for differences in food and nutrient intakes between PBM consumers (*n* = 101) and nonconsumers (*n* = 1845). Multiple regression analyses were conducted to assess PBM consumption as an independent predictor of nutrient intakes.

**Results:**

PBM consumers consumed more fruits, vegetables, and pulses (median [interquartile range]: 346 [234, 484] compared with 228 [121, 365] g/d, *P* < 0.001) and less animal meat compared with non-PBM consumers (17 [0, 105] compared with 125 [74, 185] g/d, *P* < 0.001). PBM consumers had intakes that were more in line with DRVs compared with non-PBM consumers, with higher intakes of fiber, vitamins E, A, C, and folate, calcium, magnesium, zinc, and copper (all *P* < 0.01). No differences were observed between PBM consumer groups in protein, saturated fatty acids, vitamins B12 and D, iron, potassium, iodine, and sodium intakes (all *P* > 0.05). Regression analyses confirmed that PBM consumption was independently associated with more favorable intakes of specific nutrients.

**Conclusions:**

PBM consumers had more favorable food and nutrient intakes than nonconsumers, indicating that PBM can be part of a healthier and more plant-based diet.

## Introduction

Current dietary patterns rich in animal-derived foods, especially red and processed meat, have negative impacts on climate and the environment, human health, animal welfare, and global food security [[1], [2], [3]]. Recognizing these issues, a transition toward healthier and more sustainable diets with increased consumption of plant-based foods and decreased consumption of animal-derived products is required [1,4], and recommended by global health authorities [5]. In parallel, food industry innovation has driven a rapid expansion of the plant-based meat (PBM) market, aiming to provide alternatives that align with these health and sustainability goals [6]. PBM, which mimics the taste, texture, and appearance of animal meat, could play an important role in the transition toward a more plant-based diet, as it addresses key consumer needs, such as taste and convenience, and easily fits in people’s habitual diets and meals without the need to adjust existing eating and cooking patterns [7,8]. However, concerns about the nutritional quality of PBM (e.g., lacking important nutrients from meat or high sodium content) and the level of processing that is used to make PBM are barriers for consumers to adopt PBM into their regular diet [9]. At the same time, nutritional guidance on the consumption of PBM by health authorities is lacking because of insufficient scientific data on health effects [10,11].

To date, little is known about the nutritional intake and adequacy of the diet of PBM consumers. The available evidence is mostly based on inferences made from the nutritional composition of PBM or from theoretical modeling studies. A recent systematic review found that PBM provides a range of essential nutrients and has a nutrient density comparable to animal meat, although the type and amounts of nutrients provided differ [12]. PBM is generally lower in fat and SFAs but is higher in carbohydrates, fiber, vitamin E, calcium, and manganese. In contrast, animal meat is higher in vitamins B3, B6, B12, K, selenium, and zinc, whereas the protein, sodium, and iron content of PBM and animal meat are comparable [12]. Modeling studies that investigated the effect of replacing animal meat with PBM broadly mirror these findings, though they often show a lower protein and higher sodium intake in PBM diets [[13], [14], [15], [16], [17], [18]]. Yet, these modeling studies do not represent real-life eating patterns as they generally assume a one-to-one substitution of animal meat with PBM and often simulate a substantial reduction of meat by 50% to 100%. However, in real life, people consuming PBM may make other food choices than exclusively replacing meat with PBM, which can affect overall dietary intakes. Also, consumers are more likely to decrease their consumption of animal meat rather than eliminate it entirely [19], which suggests that the reduction in intake may be less than what modeling studies predict. In addition, the outcomes of modeling studies evidently depend on the replacement products applied in the model. For example, replacing animal meat with PBM based on either mycoprotein, soy, or other legumes has been shown to have differential effects on nutrient intake and adequacy [16], and replacement with fortified PBM resulted in more adequate nutrient intakes than replacement with unfortified PBM products [16,20,21].

Empirical evidence on the nutritional quality of PBM consumers’ diets is limited and inconclusive. One observational study of a nationally representative sample of adults in the United Kingdom reported higher diet quality scores among mycoprotein consumers compared with nonconsumers [22]. Another observational study that specifically looked at vegetarians found lower but adequate energy and macronutrient intakes among vegetarian PBM consumers as compared with vegetarian non-PBM consumers [23]. A randomized controlled trial, in which participants were provided with PBM products for 4 wk, and in which PBM consumption increased, whereas animal meat consumption decreased, found no meaningful changes in the nutritional composition of the diet compared with a control group [24]. It should be noted that all these studies only considered a limited set of macronutrients and micronutrients.

More empirical research on dietary intakes of PBM consumers is needed to inform the development of dietary recommendations on PBM and improve its nutritional composition [25]. Therefore, this study aimed to assess empirical data on food consumption and explore intakes of a broad range of nutrients of PBM consumers. We compared dietary intakes with those of non-PBM consumers and with dietary recommendations. To account for personal factors that may influence dietary intakes, we conducted multiple regression analyses to assess whether PBM consumption is an independent predictor of dietary intakes. In addition, we compared dietary intakes within PBM consumers on days on which they consumed PBM compared with days on which they consumed animal meat.

## Methods

### Study design and population

We used dietary intake data of adults in the United Kingdom participating in the National Diet and Nutrition Survey (NDNS) rolling program [26]. The NDNS rolling program is a continuous, annually conducted, national cross-sectional survey in the United Kingdom, assessing dietary intake and nutritional status of a random sample of the population living in private households. Because the availability, acceptance, and consumption of PBM have increased considerably over the past decade [27,28], we only included data of the most recent 5 y of the NDNS rolling program (i.e., 2014–2019), which were available at the time the analysis was conducted (*n* = 6281). Children younger than 18 y, adults above 65 y, and pregnant and lactating women were excluded from the analysis, as well as participants with an unrealistic low or high reported mean energy intake, i.e., <2.095 MJ/d or >14.644 MJ/d (i.e., <500 kcal/d or >3500 kcal/d) for women and <3.347 MJ/d or >16.736 MJ/d (<800 kcal/d or >4000 kcal/d) for men [29]. In addition, participants who indicated they were vegetarian or vegan but reported consuming meat during the dietary assessment period were also excluded.

### Data collection

Information on participants’ sociodemographic background and lifestyle was assessed during an interview. Height (m) and weight (kg) were measured, and BMI was calculated as kg/m^2^. Dietary intake was assessed using a 4-d dietary diary completed on 4 consecutive days starting on any day of the week. Participants were asked to record all foods and drinks consumed during this period. Portion sizes were estimated based on household measures or photographs of small, medium, and large portions of frequently consumed foods as displayed in the food diary. Trained interviewers visited participants at home twice to review the food diary, clarify questions, and fill in any missing information with the participants. Dietary data were coded and categorized into main and sub food groups by trained staff and then entered into a modified version of the Medical Research Council’s dietary assessment system DINO (“Diets In Nutrients Out”) [30]. The NDNS was conducted according to the Declaration of Helsinki, and ethical approval was obtained from the Cambridge South NRES Committee (ref. no.: 13/EE/0016). More details about the methodology of the NDNS have been reported elsewhere [26].

### Classification of food groups, consumer groups, and consumption days

For our analyses, PBM was defined as all plant-based products that mimic the taste, texture, and appearance of animal meat. Other products that can substitute animal meat, such as tofu, falafel, veggie or nut burgers, or cheese-based alternatives were not considered in this definition. Also, ready meals or snacks (like pies or pastries) that contained PBM and plant-based deli meat products were excluded from our PBM definition, as their PBM content was not specified, and they contributed minimally to the overall diet due to limited use. As the classification of food groups applied by the NDNS was not specific enough for our research aims, we created 4 new food groups. These were “Plant-based meat,” which only included products that fit our definition of PBM; “All meat alternatives,” which also included alternatives that do not mimic animal meat, as well as plant-based deli meat, and ready meals and snacks containing PBM; “Beans and pulses,” which we separated out from the food group “vegetables” because of their different nutritional profile, particularly in terms of protein and micronutrients; and the overarching food group “ Fruits and vegetables,” which combines the intake of fruits, vegetables, beans, and pulses, in order to compare intakes with recommended fruit and vegetable intakes in the United Kingdom, which also includes beans and pulses [31].

Participants who reported consuming PBM on at least 1 d of the 4-d dietary assessment were considered PBM consumers. All others not consuming PBM during the 4-d dietary assessment were considered non-PBM consumers. Participants were further classified as vegetarians/vegans, flexitarians, or frequent meat eaters based on their reported food intake and consumption habits. Participants who indicated to follow a vegetarian or vegan diet during the lifestyle interview and who did not report consumption of any meat or meat products during the 4-d dietary assessment were classified as vegetarians/vegans. Due to the low number of participants following either a vegetarian or a vegan diet, participants were grouped together. Flexitarians were defined as participants who reported consuming meat or meat products on 1 or 2 d during the 4-d dietary assessment period. Finally, participants who consumed meat or meat products on 3 or 4 d during the assessment period were considered to be frequent meat eaters.

Within the group of PBM consumers, we distinguished between days on which PBM was consumed (i.e., PBM days), days on which no PBM was consumed but on which animal meat was consumed (i.e., meat days), and days on which neither PBM nor animal meat was consumed (i.e., other days).

### Nutrient intakes and dietary reference values

To calculate the dietary intake of energy, macronutrients, and micronutrients from the 4-d diaries, the food composition data from the NDNS Nutrient Databank were used. Average daily consumption of food groups and intake of energy and nutrients was first calculated for each participant across the 4 d of the dietary assessment. We then summarized intakes for PBM consumers and non-PBM consumers. Among PBM consumers, we additionally summarized day-type–specific intakes for PBM days, meat days, and other days. Total daily energy and nutrient intakes were expressed as a percentage of age- and sex-specific dietary reference values (DRVs) in the United Kingdom. If an estimated average requirement (EAR) was defined for a nutrient, we compared intakes against the EAR [[32], [33], [34]]. For nutrients for which no EAR had been established, intakes were compared against the adequate intake (AI) [32,35], reference nutrient intake (RNI) [32], or tolerable upper intake level [36]. Vitamin E intake was compared with the EAR as set by the Institute of Medicine, as no DRVs for vitamin E had been set in the United Kingdom [37]. For an overview of DRV used in the analyses, see Supplementary Table 1. A food was considered to significantly contribute to nutrient intake if it provided at least 10% of the total daily nutrient intake.

Two participants had missing data on food consumption for one of the days of the dietary assessment due to illness, but available data from the other days were included in the analysis (pairwise deletion); no imputation was performed. Intakes from supplements were not considered in the analysis.

### Statistical analysis

As the assumptions for parametric analyses (including visual inspection of residual Q–Q plots) were violated and intake data were not normally distributed, we used nonparametric tests. Differences in dietary intakes of PBM consumers and non-PBM consumers were tested with Mann–Whitney *U* tests. Differences in dietary intakes of PBM consumers on PBM days compared with meat days and PBM days compared with other days were examined using Sign tests. Results were reported as median and IQR intakes (unless indicated otherwise) along with common language effect sizes (CLESs) that describe the probability that a randomly selected observation from one group has a higher value than a randomly selected observation from the comparison group. CLES values range from 0 to 1, with 0.5 indicating no difference between groups, whereas values close to 0 or 1 indicate increasingly stronger group separation. Subgroup analyses were carried out for flexitarians, frequent meat eaters, and vegetarians/vegans.

We performed hierarchical multiple regression analyses, adjusting for personal factors that may influence dietary intakes, to examine whether PBM consumption independently predicts nutrient intakes. As covariates, we included age, sex, education, ethnicity, and area of residence for their association with PBM consumption [38,39], energy intake for its association with nutrient intakes [40], and BMI and alcohol as indicators of a healthy lifestyle. Physical activity level and smoking status were also considered as covariates but were not included due to a high proportion of missing data (i.e., ≥55%). In the first block of the regression analyses (model 1), all mentioned covariates were entered simultaneously as predictors. In the second block (model 2), PBM consumption was added as an additional predictor. Assumptions for multiple regression analyses (including visual inspection of residual Q-Q plots, homoscedasticity, collinearity, leverage, and Cook’s distance) were met for most nutrients, except fiber, iodine, niacin, phosphorus, vitamins B12, C, D, and zinc. Square root transformations resolved issues for these nutrients. Assumptions for most food groups were not met even after transformations; therefore, results of the regression analysis for food groups were not reported. Results of regression analyses were reported as β-coefficients with SEs and the change in explained variance (ΔR^2^) from model 1 to model 2 to quantify the incremental contribution of PBM consumption. Statistical significance of ΔR^2^ was assessed using a partial *F* test for nested linear models (extra sum-of-squares test). All statistical tests were 2-sided with statistical significance at *P* < 0.05. Bonferroni correction was applied by multiplying *P* values by the number of tests (19 for food groups; 28 for nutrients), capping at 1.0. Analyses were conducted in R (R Core Team) using RStudio 2026.01.0 (Build 392).

## Results

### Participant characteristics

**FIGURE 1:**
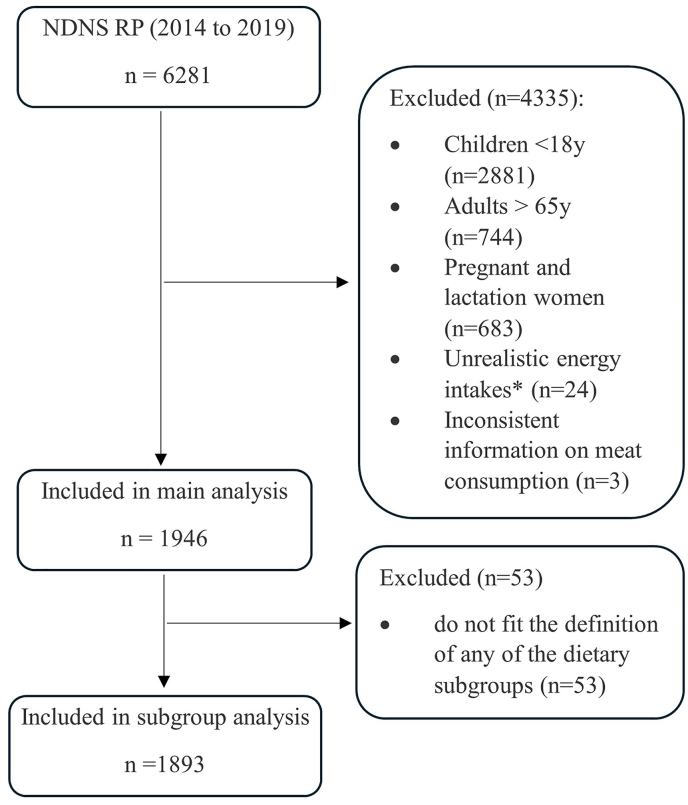
Flow chart of participants from the National Diet and Nutrition Survey Rolling Program (NDNS RP) years 2014–2019 in the United Kingdom, who were included in the analyses. ∗ Energy intake < 2.095 MJ/d or >14.644 MJ/d for women and <3.347 MJ/d or > 16.736 MJ/d for men.

A total of 1946 participants were included in the main analysis (Figure 1). For subgroup analyses, 53 participants were additionally excluded as they did not meet the criteria for vegetarians/vegans, flexitarians, or frequent meat eaters. They did not consume any meat during the dietary assessment period but did not indicate that they followed a vegetarian or vegan diet either.

Of the total sample, 5.2% were PBM consumers. The percentage of PBM consumers was considerably higher among vegetarians/vegans (46%) than flexitarians (5.8%) and frequent meat eaters (2.2%) (Table 1). Overall, PBM consumers were more educated and had a somewhat lower BMI than non-PBM consumers. Reported total energy intake was low in both PBM and non-PBM consumers (i.e., 84% and 80% of EAR, respectively).

**TABLE 1:** Characteristics of PBM consumers and non-PBM consumers (18–64 y), based on the National Diet and Nutrition Survey years 2014–2019 in the United Kingdom.

	All		Flexitarians		Frequent meat eaters		Vegetarians/vegans	
	PBM consumers (*n* = 101)	Non-PBM consumers (*n* = 1845)	PBM consumers (*n* = 21)	Non-PBM consumers (*n* = 338)	PBM consumers (*n* = 33)	Non-PBM consumers (*n* = 1449)	PBM consumers (*n* = 24)	Non-PBM consumers (*n* = 28)
Males (%)	53.5	53.2	66.7	43.5	54.5	59.1	58.3	50
Age 1	41.9 ± 13.7	44.4 ± 13.9	38.6 ± 13.6	46.6 ± 14.0	42.7 ± 13.7	43.8 ± 13.9	40.4 ± 14.3	44.9 ± 16.3
Country (%)								
England	77.2	66.2	61.9	72.5	72.7	64.4	91.7	71.4
Northern Ireland	7.9	15.8	19.0	9.5	9.1	17.8	4.2	7.1
Scotland	6.9	6.4	9.5	7.1	9.1	6.1	4.2	7.1
Wales	7.9	11.5	9.5	10.9	9.1	11.7	0.0	14.3
Educational level (%) 2								
High	48.5	29.0	33.3	31.4	48.5	27.4	54.2	57.1
Medium	22.8	24.1	19.0	18.9	27.3	25.7	16.7	7.1
Low	18.8	36.6	33.3	39.9	18.2	36.4	16.7	21.4
Other	8.9	8.5	14.3	8.3	6.1	8.5	8.3	14.3
Ethnicity (%)								
White	92.1	89.3	95.2	87.3	90.9	90.7	100.0	71.4
Mixed	2.0	0.7	4.8	0.3	0.0	0.8	0.0	0.0
Black	1.0	2.5	0.0	2.4	0.0	2.4	0.0	0.0
Asian	4.0	5.6	0.0	8.0	6.1	4.3	0.0	28.6
Other	1.0	1.4	0.0	2.1	3.0	1.2	0.0	0.0
Body mass index 1	26.0 ± 4.2	27.5 ± 5.3	25.9 ± 3.9	26.4 ± 4.8	25.9 ± 3.6	27.9 ± 5.4	25.4 ± 4.4	25.7 ± 4.1
Weight status (%) 3								
Underweight	1.0	1.3	0.0	2.1	3.0	1.0	0.0	3.6
Normal weight	38.6	30.8	38.1	37.0	33.3	29.0	45.8	39.3
Overweight	40.6	35.7	33.3	36.1	45.5	35.6	41.7	42.9
Obesity	12.9	21.7	19.0	15.1	9.1	23.5	4.2	14.3
Severe obesity	0.0	2.8	0.0	1.2	0.0	3.2	0.0	0.0
Total energy intake (MJ/d) 4	7.7 (6.5, 9.1)	7.5 (6.0, 9.2)	7.5 (6.8, 8.8)	6.6 (5.3, 8.3)	7.9 (6.3, 9.2)	7.7 (6.2, 9.4)	8.5 (6.1, 10.2)	7.1 (6.1, 8.7)
Alcohol consumption, (g/d) 4	4.7 (0.0, 18.6)	0.7 (0.0, 17.4)	0.0 (0.0, 4.6)	0.0 (0.0, 15.6)	13.4 (3.3, 27.8)	2.3 (0.0, 18.0)	1.3 (0.0, 21.5)	0.0 (0.0, 5.9)

Abbreviation: PBM, plant-based meat.

1 Values are reported as mean ± standard deviation.

2 High education: degree or equivalent; medium education: higher education, GCE A level; Low education: GCSE grades A–G, no qualifications; Other: foreign qualifications, still in full-time education.

3 Underweight, BMI (kg/m^2^) < 18.5; normal weight, BMI ≥18.5 and <25; overweight, BMI ≥25 and <30; obesity, BMI ≥30 and <40; severe obesity, BMI ≥ 40.

4 Values are reported as median (25th percentile; 75th percentile).

### PBM consumption

PBM consumers reported consuming PBM on 1.6 ± 0.9 d (mean ± SD) of the 4-d dietary assessment period, with the highest frequency in vegetarians/vegans (2.2 ± 0.9 d) and the lowest in frequent meat eaters (1.1 ± 0.3 d). Across the 4 d, the median (IQR) consumption of PBM by PBM consumers was 25 (17, 49) g per day. The consumption varied among subgroups, i.e., vegetarians/vegans had the highest median consumption of PBM (66 [36, 79] g/d), followed by flexitarians (32 [19, 42] g/d) and then frequent meat eaters (21 [10, 29] g/d) (Supplementary Table 2). On days on which PBM was eaten, median consumption was 80 [52, 117] g/d of PBM, contributing 19 [10, 25]% to total daily protein intake. Besides protein, PBM contributed considerably (i.e., ≥10%) to daily intakes of fiber, vitamin B2, niacin, zinc, phosphorus, magnesium, and sodium (Supplementary Table 3). Of the PBMs that were consumed, 77% was based on mycoprotein, 7.5% was based on soy, and the protein source was not specified for 15%. The average nutrient composition of PBM consumed, weighted for the proportion in which the products were reported to be consumed, is presented in Table 2. PBM made up 89% of all meat alternatives consumed among PBM consumers. Non-PBM consumers hardly consumed any other meat alternatives.

**TABLE 2:** Nutrient composition of plant-based meat and animal meat products as consumed, per 100 g.

	Plant-based meat		Animal meat	
	Mean	SD	Mean	SD
Energy (kJ)	534.2	213.1	827.8	272.3
Protein (g)	14.0	3.9	21.0	8.5
Fats (g)	4.8	4.4	10.2	6.8
Carbohydrates (g)	7.4	4.4	5.7	8.4
Fiber (g)	5.9	2.0	0.7	1.0
SFAs (g)	0.85	0.60	3.70	2.85
PUFAs (g)	1.59	1.55	1.41	1.16
Vitamins				
Vitamin A (μg RE)	8.92	39.9	97.2	1150
Thiamin (mg)	0.37	0.61	0.21	0.28
Riboflavin (mg)	0.41	0.53	0.17	0.21
Niacin (mg)	3.39	1.43	10.3	5.61
Vitamin B6 (mg)	0.07	0.16	0.34	0.20
Folate (μg)	23.8	12.4	10.5	29.6
Vitamin B12 (μg)	0.15	0.39	1.03	2.71
Vitamin E (mg)	0.43	0.83	0.53	0.72
Vitamin C (mg)	0.25	1.82	1.20	3.23
Vitamin D (μg)	0.07	0.24	0.43	0.34
Minerals				
Calcium (mg)	51.2	41.1	30.3	35.2
Copper (mg)	0.14	0.10	0.11	0.58
Iron (mg)	1.30	1.52	1.22	1.06
Iodine (ug)	3.67	3.52	7.72	4.94
Potassium (mg)	222	243	288	98
Magnesium (mg)	41.6	26.3	22.5	7.1
Sodium (mg)	383	181	352	387
Phosphorus (mg)	213	37	196	76
Selenium (μg)	4.3	2.3	10.6	5.4
Zinc (mg)	5.37	2.45	2.13	1.76

Abbreviation: RE, retinol equivalent.

### Dietary intake of PBM consumers compared with non-PBM consumers

#### Food consumption

**FIGURE 2:**
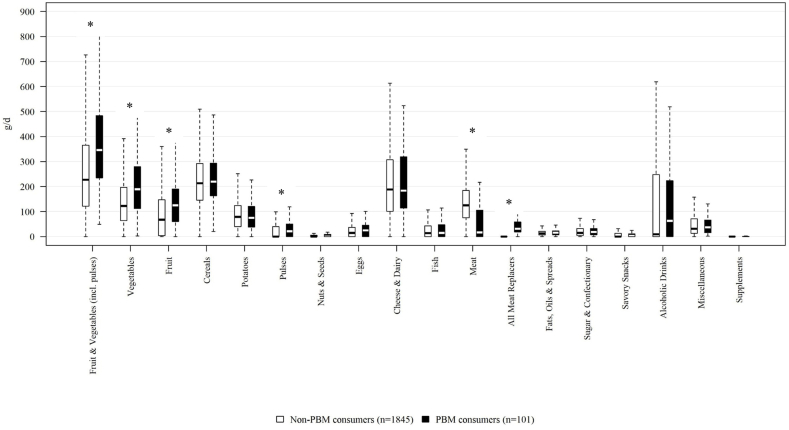
Boxplots showing median (line) and IQR of food consumption (g/d) among PBM consumers and non-PBM consumers. The bars for nonalcoholic drinks are not presented as they exceeded the range of the figure. Whiskers represent the most extreme values within 1.5 × IQR. ∗ Significant difference, Mann–Whitney *U* test (Bonferroni adjusted *P* < 0.05). PBM, plant-based meat.

PBM consumers consumed significantly more fruits, vegetables, and pulses (346 [234, 484] g/d) compared with non-PBM consumers (228 [121, 365] g/d, CLES = 0.66, *P* < 0.001). In addition, they consumed less animal meat (17 [0, 105] g/d) compared with non-PBM consumers (125 [74, 185] g/d, CLES = 0.17, *P* < 0.001) (Figure 2 and Supplemental Table 2). PBM consumers' total median fruit and vegetable consumption (including pulses) amounted to 87% of the recommended daily intake (i.e., 400 g/d) [31] compared with 57% of the recommended daily intake among non-PBM consumers. In addition, PBM consumers’ median meat consumption (17 [0, 105] g/d) was in line with UK guidelines (i.e., recommending no >70 g/d of red and processed meat) [31]. Consumption of other protein-rich foods, like fish, dairy, eggs, and nuts, seeds, did not significantly differ between PBM consumers and non-PBM consumers.

#### Energy and nutrient intakes

**FIGURE 3:**
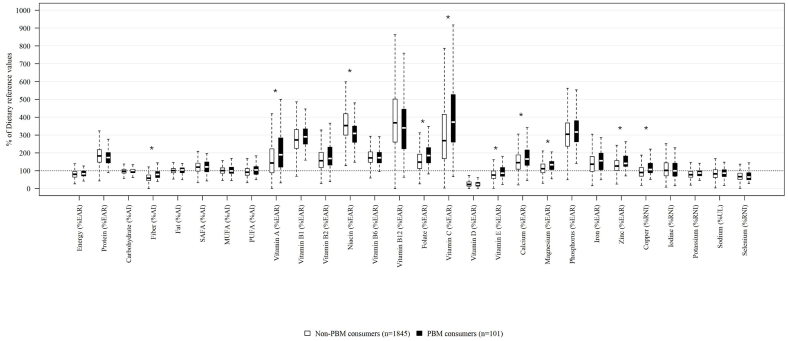
Boxplots showing median (line) and interquartile range (IQR) of daily energy and nutrient intakes of PBM consumers and non-PBM consumers, expressed as percentage of dietary reference values (dotted line). Whiskers represent the most extreme values within 1.5 × IQR. ∗ Significant difference, Mann–Whitney *U* test (Bonferroni adjusted *P* < 0.05). AI, adequate intake; EAR, estimated average requirement; PBM, plant-based meat; RNI, reference nutrient intake; UL, tolerable upper intake level.

When comparing daily nutrient intakes between PBM consumers and non-PBM consumers, intakes of fiber, vitamins A, C, E, folate, calcium, magnesium, zinc, and copper was significantly higher (all CLES >0.58, *P* < 0.01), and niacin was significantly lower (CLES = 0.32, *P* < 0.001) in PBM consumers compared with non-PBM consumers (Figure 3 and Supplementary Table 4). For the majority of these nutrients, median intakes met DRVs in both groups. However, intakes of PBM consumers were closer to DRVs for fiber (79 [61, 96]% compared with 59 [45, 76]% of AI, CLES = 0.69, *P* < 0.001) and vitamin E (86 [68, 118]% compared with 76 [57, 99]% of EAR, CLES = 0.58, *P* = 0.003) when compared with non-PBM consumers, and the median intake of copper met DRV in PBM consumers but not in non-PBM consumers (107 [88, 142]% compared with 90 [70, 117]% of RNI, CLES = 0.63, *P* < 0.001). In both groups, intakes of protein, vitamins B1, B2, B6, B12, phosphorus, iron, and iodine met DRVs, and intakes of energy, vitamin D, potassium, and selenium were below DRVs. Moreover, the intake of sodium was below and intake of SFA above the maximum target intake in both groups.

Hierarchical regression analysis showed that PBM consumption was an independent predictor of intakes of fiber (*P*_change_ < .001), niacin (*P*_change_ < 0.001), folate (*P*_change_ <0.001), vitamin C (*P*_change_ = 0.004), calcium (*P*_change_ = 0.010), magnesium (*P*_change_ = 0.001), and zinc (*P*_change_ <0.001). The observed associations between PBM consumption and nutrient intakes were generally in line with the described differences in nutrient intake between PBM consumers and non-PBM consumers. The contribution of PBM to the predictive power of the model was greatest for fiber (*R*^2^ = 0.028) and for zinc (*R*^2^ = 0.015). See Supplementary Table 5 for details of the regression analyses.

#### Subgroup analyses

When comparing dietary intakes across dietary subgroups, similar patterns in food and nutrient intake as in the main analysis were seen for frequent meat eaters and flexitarians, although few significant differences were found. The trends suggested a higher combined consumption of fruit and vegetables and a lower consumption of animal meat in PBM consumers as compared with non-PBM consumers (Supplementary Table 2). This trend was not observed among vegetarians/vegans. Regarding nutrient intakes, among flexitarians and frequent meat eaters, PBM consumers had a significantly higher fiber intake, and among vegetarians/vegans, PBM consumers had a significantly higher zinc intake compared with non-PBM consumers (Supplementary Table 4).

### Dietary intake of PBM consumers on PBM days compared with meat days

Among all PBM consumers, 49 had 1 or more meat days, 69 had 1 or more other days, and 23 had both meat and other days. Six PBM consumers consumed PBM on all 4 d of the dietary assessment (i.e., they had no meat or other days). Due to the small number (*n* = 7) of frequent meat eaters on other days, i.e., free of both animal meat and PBM, the subgroup comparison of PBM days compared with other days was limited to flexitarians and vegetarians/vegans.

#### Food consumption

Food consumption varied for PBM consumers on days when they ate PBM, animal meat (but not PBM), or neither (Table 3 and Supplementary Table 6). On PBM days, there was a trend toward a higher consumption of fruit, vegetables, and pulses (360 [241, 514] g/d) compared with meat days (280 [178, 386] g/d, CLES = 0.71, *P* = 0.07). On the other hand, on PBM days, consumption of meat (CLES = 0.10; *P* < 0.001) and alcoholic drinks (CLES = 0.26; *P* < 0.001) was significantly lower than on meat days. Notably, PBM consumers consumed considerably less PBM on PBM days (median [IQR]: 78 [45, 112] g/d) than animal meat on meat days (median [IQR]: 150 [93, 201] g/d). On days when neither animal meat nor PBM was consumed, consumption of fish was significantly higher compared with PBM days (CLES = 0.30; *P* < 0.001).

**TABLE 3:** Median and interquartile range of daily food consumption of PBM consumers on PBM days, compared with meat days or other days^1^.

	PBM days (*n* = 49, average 1.2 d)		Meat days (*n* = 49, average 2.2 d)		*P* value 2	PBM days (*n* = 69, average 1.6 d)		Other days (*n* = 69, average 1.9 d)		*P* value 2
	Median	IQR	Median	IQR		Median	IQR	Median	IQR	
Fruit and vegetables (including pulses)	359.5	273.5	280.0	207.9	0.07	412.8	322.9	320.0	349.5	0.56
Vegetables	183.0	172.5	137.8	131.7	0.84	209.0	191.5	175.2	215.1	1.0
Fruit	100.0	225.0	92.5	162.0	1.0	117.0	188.3	103.5	145.7	1.0
Cereals	261.7	213.2	184.7	129.1	1.0	256.2	200.0	184.0	194.7	1.0
Potatoes	13.1	165.0	104.0	139.3	1.0	20.0	118.2	60.0	104.6	1.0
Pulses	0.0	27.0	0.0	27.3	1.0	4.9	64.0	0.0	38.6	1.0
Nuts and seeds	0.0	0.0	0.0	4.4	1.0	0.0	13.0	0.0	15.0	1.0
Eggs	0.0	12.8	6.1	39.8	1.0	0.0	50.0	0.0	57.0	1.0
Dairy and cheese	180.0	207.1	169.0	279.6	1.0	189.8	217.9	245.5	209.3	1.0
Fish	0.0	0.0	0.0	40.0	0.13	0.0	0.0	8.2	80.0	<0.001
Meat	8.4	70.0	150.4	107.7	<0.001	0.0	0.0	0.0	0.0	<0.001
Plant-based meat	78.0	66.6	0.0	0.0	<0.001	81.1	64.0	0.0	0.0	<0.001
All meat replacers	78.0	66.6	0.0	0.0	<0.001	85.1	62.7	0.0	0.0	<0.001
Fats, oils and spreads	11.0	24.8	9.3	12.1	1.0	14.9	20.0	15.3	24.2	1.0
Sugar and Confectionary	10.0	34.1	15.9	31.0	1.0	10.0	38.0	14.6	34.7	1.0
Savory snacks	0.0	0.0	0.0	11.4	1.0	0.0	0.0	0.0	3.3	1.0
Nonalcoholic drinks	1431.0	862.0	1529.0	1001.0	1.0	1633.0	910.0	1731.0	900.4	1.0
Alcoholic drinks	0.0	0.0	66.7	326.7	<0.001	0.0	0.0	0.0	250.0	0.38
Miscellaneous	19.3	51.8	29.7	42.5	1.0	19.3	83.7	15.0	29.1	1.0
Supplements	0.0	0.0	0.0	0.0	1.0	0.0	1.0	0.0	1.0	1.0

Abbreviation: PBM, plant-based meat.

1 PBM days are days on which PBM is consumed, meat days are days on which animal meat but no PBM is consumed, and other days are days on which neither animal meat nor PBM is consumed. Two analyses were performed, i.e., one comparing PBM days with meat days including all PBM consumers who had one or more meat days (*n* = 49), and one comparing PBM days with other days including all PBM consumers who had one or more other days (*n* = 69).

2 Sign test, *P* values are Bonferroni adjusted.

#### Energy and nutrient intake

Compared with meat days, on PBM days, PBM consumers had a significantly lower intake of protein (CLES = 0.25, *P* = 0.014), vitamin B12 (CLES = 0.25, *P* = 0.014), and vitamin D (CLES = 0.18, *P* < 0.001) but a significantly higher intake of fiber (CLES = 0.78, *P* = 0.003) (Supplementary Table 7). When compared with other days, on which neither PBM nor meat was consumed, on PBM days, intakes of fiber (CLES = 0.77, *P* < 0.001) and zinc (CLES = 0.91, *P* < 0.001) were significantly higher, and vitamin B12 (CLES = 0.25, *P* < 0.001) and iodine (CLES = 0.29, *P* = 0.02) were lower.

#### Subgroup analyses

Differences in food and nutrient intake between PBM days and meat days as observed in the main analysis were generally echoed in the subgroup of frequent meat eaters (Supplementary Table 7). Among flexitarian and vegetarian/vegan PBM consumers, fewer differences in food and nutrient intakes were observed between PBM days, meat days, and other days. Intakes of fiber and zinc were consistently higher, and intakes of vitamin B12 were lower on PBM days compared with meat days or other days.

## Discussion

This study aimed to provide insights into dietary intakes of PBM consumers using empirical data and comparing these with those of non-PBM consumers and with dietary recommendations. We found that PBM consumers had a healthier dietary pattern as compared with non-PBM consumers, with a higher consumption of fruit, vegetables, and pulses and a lower consumption of animal meat, in line with food-based dietary guidelines [31]. These observations indicate that PBM does not displace other plant foods in people’s diets but is used as a replacement of animal meat. Also, nutrient intakes of PBM consumers were generally more in line with DRVs, with higher intakes of fiber, vitamin E, and vitamins A, C, folate, calcium, magnesium, zinc, and copper than non-PBM consumers. No differences in protein, SFA, vitamins B12 and D, iron, potassium, iodine, and sodium intakes were seen between consumer groups. Overall, these findings show that PBM consumers had more favorable dietary intakes than non-PBM consumers, supporting a beneficial role of PBM in the transition toward a healthier and more sustainable plant-based diet.

Our results are in line with an earlier analysis of the NDNS rolling program (2008–2017), which found that mycoprotein consumers had a better overall diet quality than nonmycoprotein consumers [22]. Corresponding to our study, this study reported higher intakes of fiber and a similar SFA intake in mycoprotein consumers compared with nonconsumers; however, it did not report micronutrient intakes. Conversely, a randomized controlled trial in the United Kingdom demonstrated no meaningful impact on dietary intakes, other than a reduction in animal meat and an increase in PBM consumption, when providing participants with a variety of PBM products for 4 wk while participants were free in all their food choices [24]. These opposing outcomes may be explained by the fact that the trial participants, who were not regular users of PBM, were randomly allocated to a PBM diet, whereas PBM consumers in observational studies, like the NDNS, consume PBM by choice. Differences in health-related motives, which are an important driver for consuming PBM [[41], [42], [43], [44]], may influence dietary intake in empirical studies and contribute to the overall healthier dietary pattern observed among PBM consumers.

Our analyses showed that the improved nutrient intakes of PBM consumers were independently associated with PBM consumption, irrespective of lifestyle and personal factors related to healthy dietary choices. However, it is important to recognize that the more favorable nutrient intakes observed among PBM consumers cannot be attributed solely to PBM, given the higher intakes of, for example, fruit and vegetables. Nevertheless, on days when PBM was consumed, it contributed meaningfully to total intakes of several key nutrients, particularly protein, fiber, zinc, and vitamin B2 (Supplementary Table 3). This highlights the nutritional value of PBM as part of a balanced diet. Beyond the positive association between PBM consumption and nutrient intake, the more favorable food choices of PBM consumers as observed in this study support the conclusion that PBM consumers have a healthier dietary pattern and are unlikely to be at increased risk of nutrient inadequacies.

Consistent with the findings of the current empirical study, findings from theoretical modeling studies that replace animal meat with PBM repeatedly reported higher fiber intakes in PBM diets [13,[15], [16], [17], [18]]. However, there were also some deviations. While modeling studies frequently reported lower protein, vitamin B12, and SFA intakes and a higher sodium intake when replacing animal meat with PBM [13,14,[16], [17], [18]], we found no such differences despite the often lower protein, vitamin B12, and SFA content and higher sodium content in PBM compared with animal meat. This discrepancy in findings may be due to the fact that modeling studies typically assume a 1-to-1 replacement of animal meat with PBM and no concomitant other dietary changes, which is different from what we observed in our study. The relatively lower consumption of PBM as compared to animal meat may have balanced out its higher sodium content. Observed differences in the consumption of other foods, such as nonsignificantly higher intakes of eggs, and fats among PBM consumers, may have compensated for the lower protein and SFA content of PBM. These differences between theoretical modeling and observational studies highlight the importance of empirical evidence in evaluating the role PBM can play in people’s real-life diet.

A major strength of this study is that it adds to the limited empirical data on dietary intakes of PBM consumers. We assessed a comprehensive range of macronutrients and micronutrients to obtain a complete picture of the nutritional quality of PBM consumers’ diets. In addition, while most studies applied theoretical modeling techniques to make inferences about the potential impact of replacing animal meat with PBM on people’s diet, our study provides insights into the actual food choices of PBM consumers and how these translate into overall nutrient intake. Such knowledge can help health authorities and policymakers to develop guidelines on PBM consumption and inform food manufacturers to optimize the nutritional composition of PBM. Most food-based dietary guidelines currently merely mention animal foods as a protein source; some also mention whole plant foods, such as legumes and nuts, but guidance on how PBM (and other meat alternatives) can fit in a healthy diet is often lacking [10,45]. Such guidance could contribute significantly to making dietary guidelines more sustainable and accessible and help consumers make healthier choices.

The study also had some limitations. Although it is based on a large nationwide study, with a considerable sample size of 1946 adults randomly recruited from the general population of the United Kingdom, the number of PBM consumers (*n* = 101) was limited. Furthermore, as dietary intake in the NDNS was assessed on only 4 d, consumers who ate PBM only occasionally were more likely to be missed out, whereas frequent consumers may be overrepresented in our study. Therefore, the results of this study are explorative and may not be nationally representative for the United Kingdom.

Although we accounted for personal factors that may influence dietary intakes, these may not have fully captured the underlying health motives of participants, as suggested by the persistent associations between PBM consumption and recommended food groups. To better understand the extent to which dietary intakes are directly related to PBM consumption, future studies should consider assessing and controlling for health motives among PBM and non-PBM consumers. Additionally, differentiating between low and high consumers, or between new and long-term PBM consumers, may help to get more comprehensive insights into how PBM consumption contributes to overall dietary quality.

Because cultural differences between countries may influence dietary choices and the overall dietary context in which PBM is consumed, the findings of this study may not be directly applicable to other countries. Also, in this study from the United Kingdom, the vast majority of PBM consumed was made from mycoprotein, whereas in other countries, PBM based on soy or wheat may be more common, which differ in nutrient composition [12,46].

PBM is a food category that is rapidly evolving in terms of available product formats and nutritional composition [47]. Therefore, we only included data of the 5 most recent years of the NDNS rolling program (i.e., 2014–2019) that were available at the time of the analysis. Still, these data may not accurately represent the current situation. Consumer demands for healthier plant-based alternatives are constantly driving the development of PBM with lower sodium and SFA content and fortification with micronutrients to address nutritional concerns. Therefore, the nutritional quality of PBM consumers to date and in the future may even further improve. Further research on the dietary quality of PBM consumers in other countries, and based on more recent intake data, is recommended.

The final limitation is that the intake data of the NDNS are based on self-reported food diaries, which are prone to underreporting [48]. Though we deliberately excluded subjects with extremely low or high average energy intakes [29] to account for implausible intakes, median daily energy intakes in our study were relatively low. As people with a higher BMI and lower education are more likely to underreport food intake [48], underreporting may have been more pronounced among non-PBM consumers. Given that people tend to selectively underreport unhealthy, energy-dense foods while healthier foods are reported more accurately [48,49], our overall finding that PBM consumers have healthier dietary patterns than non-PBM consumers seems to hold. Moreover, the use of discretionary salt was not assessed in the NDNS. While discretionary salt is often added to animal meat during cooking or at the table, PBM is usually preseasoned and does not require additional salt. This may also have led to an underestimation of sodium intake, particularly among non-PBM consumers. It is unclear to what extent these limitations may have influenced our results, but it can be hypothesized that, regarding nutrients to limit (i.e., sodium, SFA), PBM consumers have healthier dietary intakes than non-PBM consumers. Additional evidence indicating that PBM may promote a healthier diet comes from intervention studies, which have found that substituting animal meat with PBM can significantly lower total cholesterol, LDL-cholesterol, and triglyceride levels [50,51]. However, the available evidence is still limited, and further research is needed to better understand the long-term health effects of PBM.

Despite our finding that the overall nutritional adequacy of the diet was better among PBM consumers than non-PBM consumers, in both groups, we found inadequate intakes of fiber, vitamin D, vitamin E, selenium, and potassium, whereas SFA intakes were too high. Therefore, recommendations to increase the consumption of plant foods, including whole grains and vegetable oils, which provide fiber, vitamin E, and potassium, and to reduce the consumption of animal foods rich in SFA, remain relevant for the total population. Fatty fish and eggs are good sources of vitamin D, vitamin E, and selenium but also certain plant foods such as Brazil nuts, broccoli, or cabbage are rich in selenium [52] and could be included in a diverse, balanced diet. In addition, fortified foods and nutritional supplements can help to meet nutritional requirements, especially for vitamin D, which is naturally present in only a few foods and for which intakes in the general population are low [53].

In conclusion, in this explorative empirical study, we found that PBM consumers had favorable food and nutrient intakes that were more in line with dietary recommendations as compared with non-PBM consumers. PBM did not displace other nutritious plant foods in people’s diet but instead was accompanied by a reduced animal meat intake and increased fruit and vegetable intake. These findings suggest that PBM can support the transition to a healthier and more sustainable plant-based diet. More empirical research on the diet of PBM consumers is needed to build a knowledge base for the development of evidence-based dietary recommendations on PBM and to optimize its nutritional composition.

## Author contributions

The authors’ responsibilities were as follows—AJW, LB, and NN: designed the research; LB and NN: analyzed data and wrote the article; AJW and SB: reviewed the article and supervised the project; and all authors: read and approved the final manuscript.

## Data availability

NDNS data can be requested via the United Kingdom data service: https://datacatalogue.ukdataservice.ac.uk. Data described in the manuscript, code book, and analytic code will be made available upon request.

## Declaration of Generative AI and AI-assisted technologies in the writing process

During the preparation of this work, the authors used Microsoft Copilot (Microsoft 365 Copilot) to assist with language editing (i.e., improving the clarity, consiness and flow) of selected text passages. After using this tool, the authors reviewed and edited the content as needed and take full responsibility for the content of the publication.

## Funding

This research was funded by Unilever Foods Innovation Centre Wageningen, The Netherlands. Researchers employed by Unilever were involved in the study design, data analysis, interpretation of the findings, and preparation of the manuscript. The UK National Diet and Nutrition Survey was funded by the Office for Health Improvement and Disparities (OHID) and the UK Food Standards Agency (FSA).

## Conflict of interest

NN and AJW are employees of Unilever Food Innovation Centre Wageningen. Unilever markets food products, including plant-based meat. At the time of manuscript submission, Unilever had demerged its plant-based meat business. LB and SB declare no interests.
